# Novel Insights into the Adipokinome of Obese and Obese/Diabetic Mouse Models

**DOI:** 10.3390/ijms18091928

**Published:** 2017-09-08

**Authors:** Birgit Knebel, Simon Goeddeke, Gereon Poschmann, Daniel F. Markgraf, Sylvia Jacob, Ulrike Nitzgen, Waltraud Passlack, Christina Preuss, Hans-Dieter Dicken, Kai Stühler, Sonja Hartwig, Stefan Lehr, Jorg Kotzka

**Affiliations:** 1 Institute of Clinical Biochemistry and Pathobiochemistry, German Diabetes Center, Heinrich-Heine-University Duesseldorf, Leibniz Center for Diabetes Research, Aufm Hennekamp 65, 40225 Dusseldorf, Germany; bknebel@ddz.uni-duesseldorf.de (B.K.); simon.goeddeke@ddz.uni-duesseldorf.de (S.G.); sylvia.jacob@ddz.uni-duesseldorf.de (S.J.); ulrike.nitzgen@ddz.uni-duesseldorf.de (U.N.); waltraud.passlack@ddz.uni-duesseldorf.de (W.P.); sonja.hartwig@ddz.uni-duesseldorf.de (S.H.); jkotzka@ddz.uni-duesseldorf.de (J.K.); 2 German Center for Diabetes Research (DZD), Partner Duesseldorf, 40225 Duesseldorf, Germany; daniel.markgraf@ddz.uni-duesseldorf.de (D.F.M.); christina.preuss@ddz.uni-duesseldorf.de (C.P.); 3 Molecular Proteomics Laboratory, Biomedizinisches Forschungszentrum (BMFZ), Heinrich-Heine-University Duesseldorf, 40225 Duesseldorf, Germany; gereon.poschmann@hhu.de (G.P.); kai.stuehler@hhu.de (K.S.); 4 Institute of Clinical Diabetology, German Diabetes Center at the Heinrich-Heine-University Duesseldorf, Leibniz Center for Diabetes Research, 40225 Duesseldorf, Germany; 5 Multimedia Center, Heinrich-Heine-University Duesseldorf, 40225 Duesseldorf, Germany; hans-dieter.dicken@uni-duesseldorf.de; 6 Institute for Molecular Medicine, University Hospital Duesseldorf, Heinrich-Heine-University Duesseldorf, 40225 Duesseldorf, Germany

**Keywords:** primary adipocyte, mass spectrometry, healthy adipose tissue, diacylglycerol, diabetes and obesity

## Abstract

The group of adipokines comprises hundreds of biological active proteins and peptides released from adipose tissue. Alterations of those complex protein signatures are suggested to play a crucial role in the pathophysiology of multifactorial, metabolic diseases. We hypothesized that also the pathophysiology of type-2-diabetes is linked to the dysregulation of the adipocyte secretome. To test this, we investigated mouse models with monogenic defects in leptin signaling which are susceptible to adipositas (C57BL/6 Cg-Lep^ob^ (obob)) or adipositas with diabetes (C57BL/KS Cg-Lepr^db^ (dbdb)) according to their genetic background. At the age of 17 weeks, visceral fat was obtained and primary murine adipocytes were isolated to harvest secretomes. Quantitative proteome analyses (LC-ESI-MS/MS) identified more than 800 potential secreted proteins. The secretome patterns revealed significant differences connected to the pathophysiology of obese mice. Pathway analyses indicated that these differences focus on exosome modelling, but failed to provide more precise specifications. To investigate the relationship of secretome data to insulin sensitivity, we examined the content of diabetogenic lipids, i.e., diacylglycerols (DAGs), identified as key players in lipid-induced insulin resistance. In contrast to obob mice, fat tissue of dbdb mice showed elevated DAG content, especially of DAG species with saturated fatty acid C16:0 and C18:0, while unsaturated fatty acid C16:1 were only changed in obob. Furthermore, DAG signatures of the models specifically correlate to secreted regulated adipokines indicating specific pathways. In conclusion, our data further support the concept that the fat tissue is an endocrine organ that releases bioactive factors corresponding to adipose tissue health status.

## 1. Introduction

Obesity is a worldwide health burden caused by increased energy intake and sedentary lifestyle. It increases the overall risk for life threatening comorbidities including cardiovascular risk, hypertension, pulmonary obstructive syndrome, dyslipidemia, metabolic syndrome or diabetes, and cancer [1,2,3]. Adipose tissue comprises mature adipocytes, preadipocytes and various invasive immune cells which, in sum, act as secretory organ of bioactive proteins, designated as adipokines. The secreted adipokine patterns in a certain metabolic conditions or stage of obesity are thought to reflect the state of the adipose tissue condition and “health” or its “metabolic flexibility” [4].

A recent investigation described the secretome of visceral adipose tissue from two closely related, well-characterized and metabolically healthy mouse strains, i.e., C57BL/Ks (BKS) and C57BL/6 (C57) by combining state-of-the-art protein identification and quantification tools [5]. A reference map comprising about 600 adipokines was generated (http://www.diabesityprot.org). Both commonly used experimental “wild-type” mouse strains differ in their response to metabolic stress. In contrast to animals with C57 genetic background, mice with a BKS genetic background are prone to develop diabetes under such conditions [6,7]. Therefore, mouse models with genetic defects in leptin signaling are obese (obob; loss of function mutation in leptin) or obese and diabetic (dbdb; loss of function mutation in leptin receptor), depending on genetic background.

Obesity is accompanied by disturbed lipid metabolism, elevated levels of free fatty acids (FFA) and triglycerides (TG), either due to over-nutrition or increased hepatic de novo lipid synthesis [8]. Besides adipose tissue, multiple organs, e.g., liver, skeletal muscle, pancreas, or kidneys, are affected. These organs can be the target of ectopic lipid accumulation and obesity-associated insulin resistance. The systemic overflow with increased fluxes of plasma FFA and TGs towards these tissues leads to the ectopic accumulation of lipids and obesity associated insulin resistance, ultimately altering tissue glucose metabolism and affecting blood glucose clearance. Ectopic lipid accumulation is accompanied by the accumulation of bioactive metabolites, e.g., diacylglycerol (DAG), in the various tissues [9]. DAGs are a result of several metabolic fluxes, including triglyceride hydrolysis, triglyceride synthesis, or phosphoinositide hydrolysis. In liver, it has been shown that DAG content is significantly increased in lipid-induced hepatic insulin resistance. DAGs act as second messengers activating members of novel protein kinase C (nPKC) family [10] and the role of DAGs in Golgi/ER vesicular transport is conserved. This raised our hypothesis that an excess of these bioactive metabolites alters the intracellular signaling also in adipose tissue and in consequence, the inter-organ communication in form of the adipocyte secreted protein patterns.

We intended to investigate the specific differences in the secretome of adipocytes in states of obesity and obesity with diabetes. For this, we utilized obese and obese/diabetic mouse models to compare the adipocyte-derived, not fat tissue, secretion pattern of adipokines and return the information to the adipocyte-derived DAG patterns. Our results suggest that DAG-signaling in adipose tissue acts as intermediary between healthy or diabetic state.

## 2. Results and Discussion

**Figure 1:**
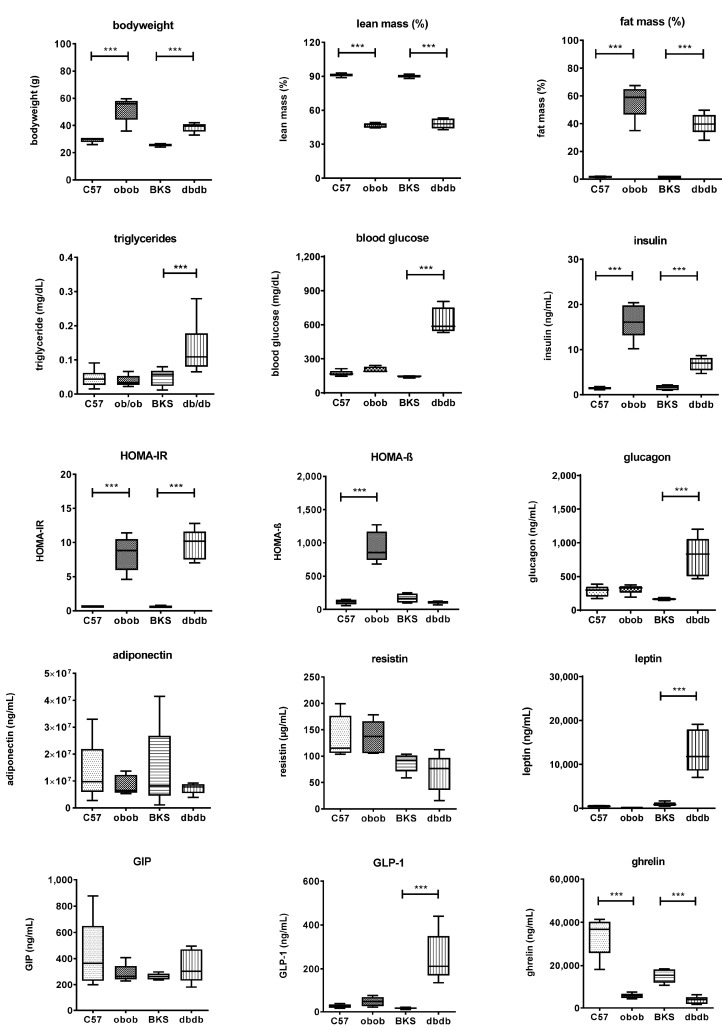
Metabolic characterization of C57, BKS, obob and dbdb mice used in the study. Data are expressed as mean ± SD (*n* = 8 of each phenotype). *** *p* < 0.001 by Student’s *t* test.

C57BL/KS.Cg-Lepr^db^ (dbdb) mice on C57BL/KS (BKS) genetic background, a well-accepted mouse model of hyperphagia induced obesity with overt diabetes, and C57BL/6.Cg-Lep^ob^ (obob) mice on C57BL/6 (C57) background which are protected from diabetes [6,7,11,12,13,14,15] were selected for this study. The clinical characteristics of the investigated mouse models are summarized in Figure 1. The obese mouse model and the obese/diabetic mouse model showed increased body weight with more than 50% fat mass compared to the lean models. Overall, the models also showed specific differences in direct comparison to the genetic background model. Fasting glucose, triglycerides, insulin, and HOMA-IR were each significantly higher in dbdb compared to BKS mice, indicating the definitions of overt diabetes in the dbdb model compared to obob mice. In contrast, in obob mice, insulin and HOMA-IR were significantly higher compared to C57. HOMA-B were strongly elevated while glucose was normal indicating that the β cells are still capable to compensate required insulin levels. Leptin was significantly elevated only in dbdb, as expected from the genetic defect of this model. Similarly, glucagon and glucagon like peptide (GLP)-1 were significantly increased only in dbdb mice. This can be attributed to the diabetic state of these mice, which was confirmed by high HOMA-IR index and thus peripheral insulin resistance. Ghrelin was 4-fold reduced in the obese and 2-fold reduced in obese/diabetic mice, whereas glucose-dependent insulinotropic peptide (GIP), adiponectin or resistin levels did not differ significantly between models.

To further analyze differences between adipose tissue of these mouse models, we determined the content of secreted proteins by mass spectrometry. The overall comparison is given in Supplementary Materials Table S1. According to our experimental design, identified proteins were located outside of intact primary adipocytes. Proteins traffic through the secretory pathway according to their N-terminal signaling sequence to reach their intracellular destination, e.g., an organelle, or to ultimately be secreted. Transport throughout the endomembrane system occurs via the endoplasmic reticulum and the golgi apparatus towards the plasma membrane. Here, the release can occur passive, active channel-mediated, or driven by formation of secretory granules and exosomes. Proteins are targeted according to classical (SP(+)) or non-classical (SP(−)) signal sequences. Proteins without any known signal sequence (NP) are thought to follow e.g., pore-mediated translocation across the plasma membrane, ABC transporter-based secretion or autophagosome/endosome-based secretion [16]. This classification can also help to determine transmembrane proteins [17]. Nevertheless, we cannot completely exclude that some of the proteins might be identified due to apoptosis or autophagy.

The comparisons identified 873 non-redundant proteins. Of these, 216 were assigned to contain a SP(+) signal peptide, 290 were SP(−), thus not carrying a classical signaling peptide and 367 were NP without a signaling domain. The NP proteins contain 164 proteins, which can be assigned to mouse adipocyte exosomes. Nevertheless, we cannot completely role out that some of the identified proteins were derived from autophagy or apoptosis in vitro cultured primary adipocytes after isolation from adipose tissue. The pairwise comparison of the four animal models showed significant alteration (Supplementary Materials Table S2). The comparisons of BKS and dbdb models identified 198 upregulated and 153 downregulated proteins in dbdb (94 SP(+), 118 SP(−), 139 NP). The comparisons of C57 and obob indicated 182 upregulated and 118 downregulated proteins in obob (88 SP(+), 98 SP(−), 114 NP). The individual comparisons indicated in the lean models 108 upregulated and 136 downregulated proteins in BKS (59 SP(+), 87 SP(−), 98 NP), and in the obese and obese/diabetic models 88 upregulated and 112 downregulated proteins in dbdb (61 SP(+), 75 SP(−), 64 NP). Table 1 (see Supplementary Materials Table S2 for complete analyses) summarizes the top 10 up- and down- regulated putative secreted (SP(+), SP(−)) proteins of the comparisons. Furthermore, there were proteins specific for either genotype in the comparisons (Table 2). Additionally, there where 38 solitaire proteins in BKS vs. C57 (8 SP(+), 13 SP(−), 17 NP), 22 in C57 vs. obob (10 SP(+), 6 SP(−), 6 NP), 60 in BKS vs. dbdb (17 SP(+), 17 SP(−), 26 NP) or 10 in dbdb vs. obob (5 SP(+), 2 SP(−), 3 NP).

**Figure 2:**
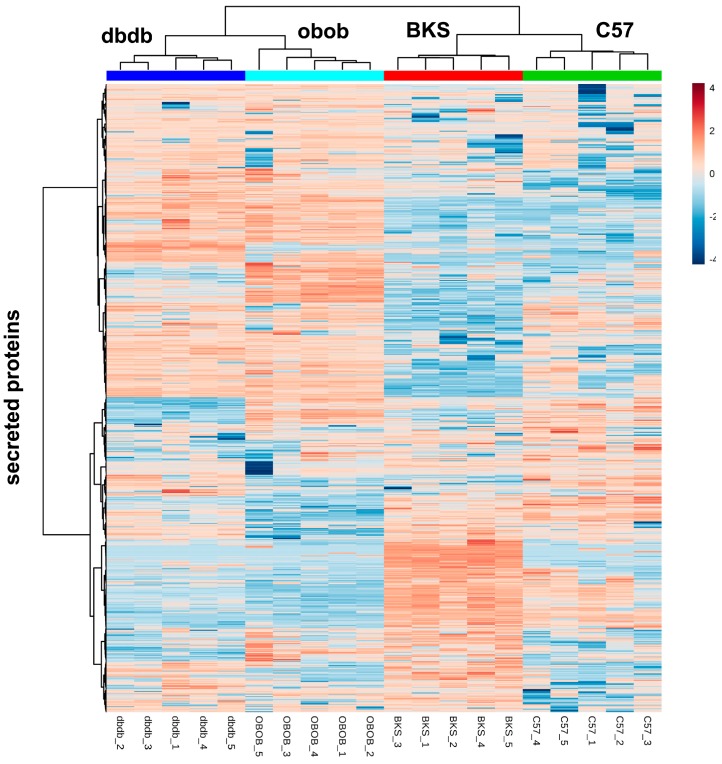
Heatmap of all identified adipokines.

**Figure 3:**
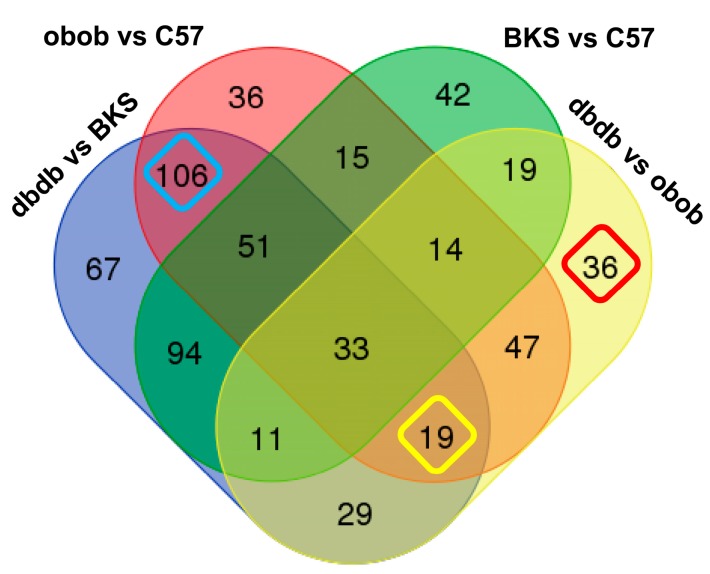
Venn analyses of differential abundant proteins. Proteins with differential abundance in the comparisons C57 vs. BKS, C57 vs. obob, BKS vs. dbdb and dbdb vs. obob (>1.5 fold, one-way ANOVA posthoc *p*-value < 0.05) were analyzed for overlap to determine genotype specific and genotype independent alterations. Genotype independent differential abundant proteins for “obesity” (*n* = 106, turquoise), diabetes (*n* = 36, red) and diabetes despite obesity (*n* = 19, yellow) are highlighted. *p*-Value was determined by Welch test. Further information of proteins of all groups are detailed in Supplementary Materials Table S3.

With regard to function, top regulated proteins or solitaire proteins were comprehensibly, among them proteins involved in lipid transport (e.g., ApoE, ApoA4), enzymes (e.g., Lpl, Aad9, Acadvl, Fbp1, Acyl, Ca4, Khk, Pgp), and signaling proteins (e.g., Il6, Sdpr, Gc, Esp15, Rbp-1, Cxcl-5, -3, -9) proteins. Overall, the total adipocyte secreted proteins were able to differentiate lean, obese and the obese/diabetic mouse models (Figure 2). Nevertheless, patterns do not only show overlap according to the lean or obese and obese/diabetic phenotype, but also according to genotype. So, we compared all differentially abundant proteins in the various groups (Figure 3, Supplementary Materials Table S3). With these analyses, we were able to account on any different abundance in conditions depending on genetic background. So, we identified 36 proteins that were solely differential abundant within lean and obese mice (C57 vs. obob), 67 proteins that differed in the comparison of lean to obese/diabetic mice (BKS vs. dbdb), and 42 proteins that differed in the lean background strains. These candidates might be of interest in regard to the phenotype, but still contain the genotype bias.

Consistent with the experimental design of adipocyte secretome analyses, all comparisons in databases such as GO, KEGG or IPA annotated to keywords like “extracellular exosome” (FDR = 1.03 × 10^10^–7.13 × 10^27^), or “membrane-bounded vesicle” (FDR = 1.03 × 10^10^–7.08 × 10^24^) with the highest significance. Other keywords were rather unspecific e.g., “amide metabolism” (*n* = 7, FDR = 2.02 × 10^3^), or “regulation of protein metabolic process” (*n* = 13, FDR = 2.02 × 10^3^) for C57 based comparisons. BKS based comparisons also identified general metabolic pathways like “metabolic process” (*n* = 46, FDR = 2.86 × 10^5^), “regulation of protein transport” (*n* = 10, FDR = 7.30 × 10^4^), or “protein metabolic process” (*n* = 23, FDR = 8.94 × 10^4^).

The analyses further identified proteins, that differed in both obese and obese/diabetic models compared to the lean mice (*n* = 106, “obesity pattern”). These proteins were related to the obese phenotype independent of genotypes investigated. Another 19 proteins differed between obese and obese/diabetic regardless of genotype, and 36 proteins were specific for diabetes despite obesity as they differ among obese and obese/diabetic (Figure 3, Supplementary Materials Table S3). In pathway analyses of these protein sets, functional annotation only indicated direct secretion or vesicle secretion, as expected from experimental design (Supplementary Materials Table S3). Functional annotation identified key words like “extracellular exosome” (*n* = 70, FDR = 3.26 × 10^41^) or “membrane-bounded vesicle” (*n* = 75, FDR = 3.36 × 10^41^) for the obesity pattern, “extracellular region” (*n* = 13, FDR = 3.34 × 10^5^) for the diabetes pattern or “extracellular exosome” (*n* = 24, FDR = 5.89 × 10^13^), and “membrane-bounded vesicle” (*n* = 25, FDR = 3.96 × 10^12^) for the diabetes despite obesity pattern as best hits. Other key terms of potential interest to metabolic energy balance showed lower significance and limited numbers of assigned proteins e.g., mitochondria (BKS vs. dbdb, *n* = 17, FDR = 1.76 × 10^3^; diabetes despite obesity, *n* = 15, FRD = 6.44 × 10^7^), lipid metabolism (BKS genotype based differences, *n* = 11, FDR = 1.12 × 10^3^), lipid catabolic process, lipid- or phospholipid binding (obesity pattern, *n* = 5, FDR = 3.17 × 10^3^; *n* = 12, FDR = 4.68 × 10^4^; *n* = 9, FDR = 4.68 × 10^4^), or fatty acid degradation (diabetic despite obesity pattern, *n* = 3, FDR = 1.42 × 10^4^) (Supplementary Materials Table S3).

In general, enrichment analyses were used to facilitate the interpretation of numerous genes or proteins which are the usual outcome of hypothesis generating experimental designs. Thus, the accumulation of candidates with known biological function or interaction, either directly experiment proven or deduced from literature, were monitored in a dataset. Knowledge based pathway annotation or gene enrichment analyses can help to classify “Omics” data, but also bares some restrictions. Next to bioinformatics, the main issue being intrinsic to the experimental setting [18]. We use secreted proteins, so enrichment of secreted proteins or related pathways with highest significance confirmed our experimental approach. The other bias, for sure is the limited number of differential proteins in the specific regulations we focus on, which hampers annotations in a general way.

So, we decided to focus on our initial working hypothesis, i.e., to identify alterations in the adipocyte “communication” with regard to specific physiological states. Adipose tissue controls systemic energy storage and needs to expand in regard to metabolic needs. In healthy conditions, this can be due to hyperplasia, but in metabolically affected adipose tissue as in obesity or diabetes, increased ad libitum storage of fatty acids occurs, even in non-adipose tissues. Increased lipid load in these cells favors accumulation of fatty acids (FA)-derived metabolites such as fatty acyl-CoA or DAG which initiated cellular processes via PKC signaling [19]. As chronical process, the cells get insulin resistance with dysfunctional mitochondria resulting in the development of obesity and diabetes. With regard to adipocyte function, the combination of both should alter the DAG patterns in adipocytes, like observed in other insulin-sensitive tissues as liver, skeletal muscle or even pancreas [9,20,21,22].

**Figure 4:**
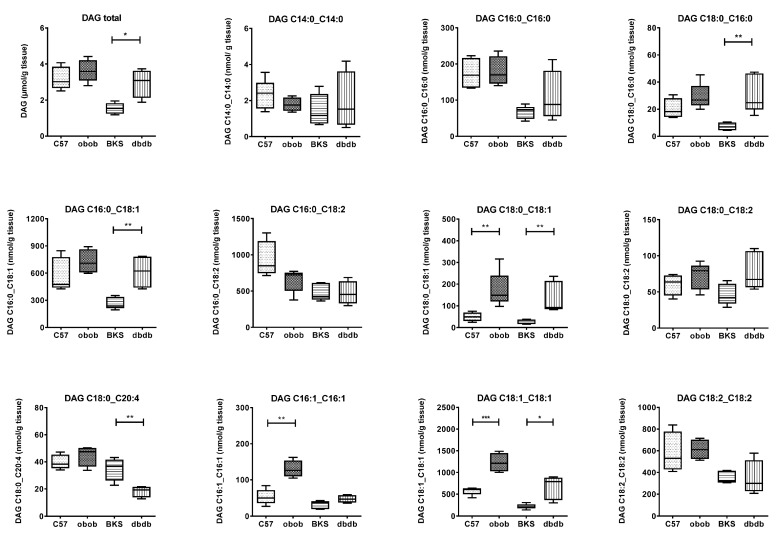
Diacylglycerol pattern in adipose tissue of C57, BKS, obob and dbdb mice. Data are given in (μmol or nmol/g tissue) and expressed as mean ± SD (*n* = 5 of each phenotype). * *p* < 0.05, ** *p* < 0.01, *** *p* < 0.001 by Student’s *t* test.

Adipose tissue DAGs were determined by mass spectrometry (Figure 4). In contrary to the obese obob, there was an increase in total DAG content in obese/diabetic dbdb mice compared to their backgrounds. Nevertheless, the comparison of the distinct DAGs revealed that in both obese models the DAG species with the fatty acid C18:1 were equally regulated (C18:0_18:1; C18:1_18:1). DAG species with saturated fatty acid C16:0 and C18:0 were only changed in dbdb (C16:0_C18:0; C16:0_C18:1, C18:0_C20:4), whereas the DAG species with unsaturated fatty acid C16:1 were only changed in obob (C16:1_C16:1).

**Figure 5:**
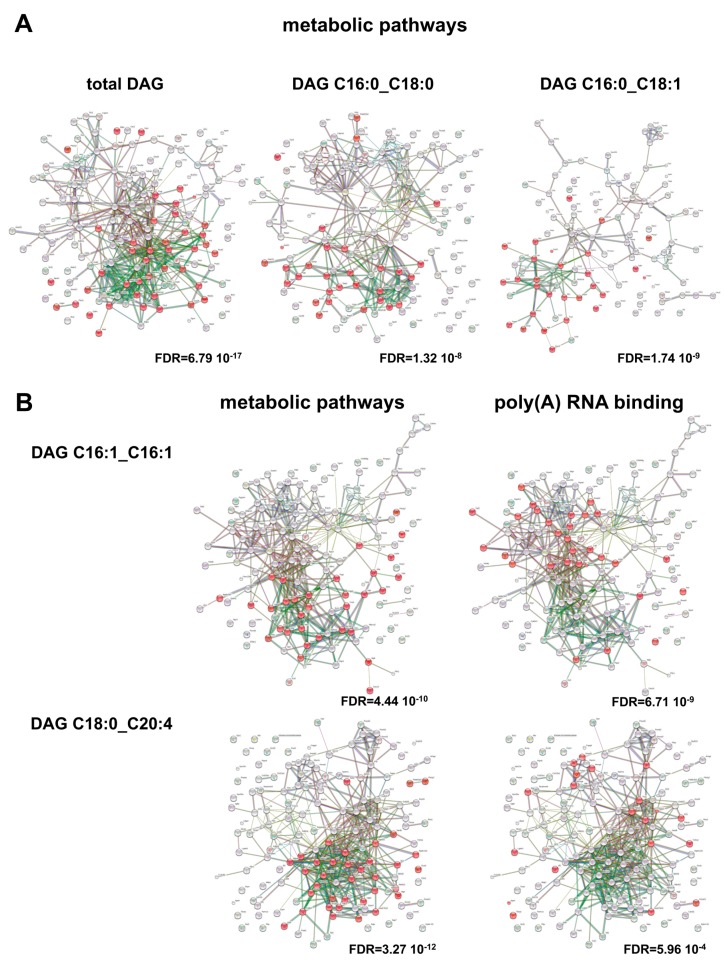
Functional network of adipokines correlated to DAG species. Adipokines with significant correlation to total DAG content or indicated DAG species were used for over representation analyses. (**A**) Adipokines correlated to total DAGs, DAG C16:0_C18:0 DAG C16:0_C18:1 species are enriched in metabolic pathways (highlighted in red); (**B**) Adipokines correlating to DAG C16:1_C16:1 and DAG C18:0_C20:4 are enriched in metabolic pathways or poly(A) RNA binding (highlighted in red). Enrichment FDR is given. Graphs show results of interaction analyses (https://string-db.org).

According to our hypothesis, intensities of a vast amount of adipose secreted proteins correlated to the total adipose derived DAGs (*n* = 152; 23 SP(+), 53 SP(−), 76 NP). Of these 105 proteins (20 SP(+), 36 SP(−), 49 NP) also showed differential abundance in either comparison of mouse phenotypes. In addition, specific DAG species correlate to adipocyte-secreted proteins (Figure 5, Supplementary Materials Table S4). Here, all secreted correlated proteins can be assigned to metabolic active proteins with the highest prevalence. Of note, DAG species specific for obob shows poly(A) RNA binding proteins as highest annotation (poly(A)RNA binding, *n* = 30, FDR = 6.71 × 10^9^, RNA binding, *n* = 31, FDR = 4.10 × 10^7^). This is also observed, if not in highest position with DAG species specific for dbdb (*n* = 20, FDR = 5.96 × 10^4^) (Figure 5). This is of interest as it focused the differences in obesity and obesity/diabetes to the concept of moonlighting enzymes in metabolic control. Moonlighting proteins or gene sharing defines various functions of a certain gene and are independent to alternative splicing, posttranslational modification or multifunctionality. Especially ancestral and conserved proteins in central metabolic processes show moonlighting functions, e.g., glycolysis or tricarboxylic acid cycle enzymes [23]. This process of metabolic regulation can account for expression levels, differential localization, protein interactions and is mediated by binding of RNA species to a distinct, but not necessary active domain of an enzyme. Best known examples of metabolic enzymes regulated by binding of RNA species are GOT2, FASN, or GAPDH [24]. We identified Adk, Aldh6A1, Aldoa, Eno, Lta4h and Hsd17B10 to be secreted from adipocytes and to correlate to DAG species C16:1_C16:1 or DAG species C18:1_C20:4. All of these proteins were previously identified in RNA interaction studies and implicated to have moonlighting functions [24]. For example, the metabolic enzyme fructose-1,6-bisphosphate aldolase (Aldoa) which catalyzes the reversible cleavage of fructose-1,6-bisphosphate to glyceraldehyde 3-phosphate and dihydroxyacetone phosphate in glycolysis and gluconeogenesis pathways, has been shown to regulate insulin-dependent glucose transporter GLUT4 in mouse adipocyte cell lines 3T3-L1 [25]. Furthermore, enolase (Eno) catalyzes the dehydrolyzation of 2-phospho-d-glycerate to phosphoenolpyruvate in glycolysis, but has also been shown to bind plasminogen and to mediate its cell surface peptidase activity [26,27]. So, one could speculate that alterations in such regulatory processes might interfere with the subcellular localization and trafficking of proteins, depending on which functions is favored, and are also an essential target in the overall picture of metabolic regulation.

## 3. Materials and Methods

### 3.1. Mouse Models

C57BL/6 (C57), C57BL/KS (BKS), C57BL/KS.Cg-^Leprdb^ (dbdb) and C57BL/KS.Cg-^Lepob^ (obob) mice were bred and maintained in a regular 12 h light/dark cycle under constant temperature, humidity (22 ± 1 °C, 50 ± 5% humidity), with free access to water and standard laboratory food (Ssniff, Soest, Germany). Mice were sacrificed by CO_2_ asphyxiation at 17 weeks of age. Mice (*n* = 5 each genotype, males) were sacrificed at 7 a.m. after 6 h food restriction and visceral adipose tissue was removed [5,28]. Adipose tissue was either processed directly to isolate adipocytes or was snap frozen in liquid nitrogen and stored at −80 °C for lipid analyses. Serum was collected by left ventricular punctuation. The Animal Care Committee of the University Duesseldorf approved all animal care and procedures (Approval#50.05-240-35/06, August 2006).

### 3.2. Metabolic Characterization of the Mouse Models

Blood parameters were measured at 17 weeks of age (*n* = 8). Blood glucose was measured with Freestyle™ and leptin, insulin as well as glucagon levels were determines using quantitative Bio-Plex Pro Mouse Diabetes 8-Plex Assay (Bio-Rad, Munich, Germany) according to the manufacturer’s instructions. Data were collected and analyzed using a BioPlex 200 instrument equipped with BioManager analysis software (Bio-Rad). To determine insulin resistance and pancreatic β cell function the surrogate parameters HOMA-IR (homeostatic model assessment of insulin resistance) and HOMA-β (homeostatic model assessment of β cell function) were used. Body composition was measured using nuclear magnetic resonance (*n* = 9–23/per genotype, Whole Body Composition Analyzer; Echo MRI, Houston, TX, USA).

### 3.3. Secretome Profiling by Liquid Chromatography (LC)-Electrospray Ionization (ESI)-MS/MS and Data Analyses

Murine mature adipocytes from visceral fat isolated by collagenase digestion were cultured for 24 h (DMEM/F12 without FCS supplementation (Thermofisher Scientific, Darmstadt, Germany)), and secretomes were harvested as described [29]. Data of all mouse models were acquired in parallel as described in detail [5]. In brief, secretome samples were tryptically digested and analyzed using LC-ESI mass spectrometry using an Ultimate 3000 Rapid Separation liquid chromatography system (Dionex/Thermo Scientific, Idstein, Germany). Afterwards, mass spectrometry was carried out (Orbitrap Elite high resolution instrument, Thermo Scientific, Bremen, Germany). For the comparison of mouse strains, log2 PSM values were used. MaxQuant (version 1.4.1.2, Max Planck Institute for Biochemistry, Munich, Germany) was used for protein and peptide identification and quantification with default parameters if not otherwise stated. Searches were carried out using 16.671 mouse sequences from the Swiss-Prot part of UniProtKB (release 9.7.2014) applying the following parameters: mass tolerance precursor (Orbitrap): mass tolerance precursor: 20 ppm firt search and 4.5 ppm after recalibration (Orbitrap), mass tolerance fragment spectra: 0.4 Da (linear ion trap), trypsin specific cleavage (maximum of one missed cleavage site), fixed modification: carbamidomethyl, variable modifications: methionine oxidation and N-terminal acetylation. For peptide and protein acceptance, the false discovery rate (FDR) was set to 1%, only proteins with at least two identified peptides were used for protein assembly. Quantification was carried out using the label-free quantification algorithm implemented in MaxQuant using a minimal ratio count of 2 and the “match between runs” option enabled.

### 3.4. Lipid Analysis of Adipose Tissue

Extraction, purification and analysis of DAGs from frozen adipose tissue samples was conducted using an LC-MS/MS approach [21]. In brief, 20 mg of adipose tissue was homogenized in 20 mM Tris/HCL, 1 mM EDTA 0.25 mM EGTA, pH 7.4, using a tight-fitting glass douncer (Wheaton Lab Supplies, Birmingham, UK). Internal standard (d517:0-DAG; Avanti Polar Lipids, Alabaster, AL, USA) was added and lipids were extracted according to Folch et al., [30]. Diacylglycerols were separated from triglycerides using solid phase extraction (Sep Pak Diol Cartridegs; Waters, Milford, MA, USA). The resulting lipid phase was dried under a gentle flow of nitrogen and re-suspended in methanol. Diacylglycerols were separated using a Phenomenex Luna Omega column (1.6 µm 100 A; Phenomenex, Torrance, CA, USA) on an Infinity 1290 HPLC system (Agilent Technologies, Waldbronn, Germany) and analyzed by multiple reaction monitoring on a triplequadrupole mass spectrometer (Agilent 6495; Agilent Technologies), operated in positive ion mode.

### 3.5. Prediction and Annotation of Secretory Proteins

Secretory protein prediction and functional annotation was done using different independent methods. First, protein information of all identified proteins was extracted from the Swiss-Prot database (http://www.uniprot.org/). To assess secretory properties, protein sequences were analysed by SignalP 4.1 [17]; (http://www.cbs.dtu.dk/services/SignalP/), SecretomeP 2.0. [31]; (http://www.cbs.dtu.dk/services/SecretomeP/) and Exocarta (http://www.exocarta.org/) [32]. Literature screening was performed with NCBI/Pubmed (http://www.ncbi.nlm.nih.gov/pubmed) and protein-protein interaction analyses with https://string-db.org.

### 3.6. Web-Based Functional Annotation

The identification types were uniprot swissprot accession or gene ID, respectively. Information driven analyses including functional annotation was performed with String v10.5 (https://string-db.org) [33], David Bioinformatics Resources 6.8 (https://david.ncifcrf.gov) [34,35], and IPA (Ingenuity^TM^, Qiagen, Hilden, Germany). For differential protein sets expression analyses, expression fold change (1.5×) and expression differences (*p*-value < 0.05) were analyzed following the core analyses modules. Differential abundant proteins (1.5× fold difference, *p*-value < 0.05 (one-way ANOVA, post hoc) were analyzed separately for C57 vs. BKS, C57 vs. obob, BKS vs. dbdb and dbdb vs. obob.

### 3.7. Statistical Methods

Statistical analyses were performed in GraphPad Prism 5.0 (GraphPad Software, Inc., San Diego, CA, USA) and SPSS 22 (IBM, Armonk, NY, USA). Data are given as mean ± standard deviation (SD) and data were directly compared with an unpaired Student’s *t* test. Figure legends indicate the statistical tests applied for each experiment in detail.

## 4. Conclusions

We showed that genetic mouse models, which are susceptible to obesity or obesity/diabetes according to their genetic background genotype show phenotype-specific differences in primary adipocyte adipokinome in quantitative proteome analyses. Knowledge based annotation of identified differentially regulated adipokinome did not add much further information. According to the predictive value of DAG-species for lipid metabolism and insulin resistance in liver and skeletal muscle [9], we determined DAG levels also as classifying parameter for lipid metabolism and insulin resistance in adipose tissue. Adipose tissue DAG patterns differ in obesity and obesity/diabetes especially of DAG species with saturated fatty acid C16:0 and C18:0 in diabetes and unsaturated fatty acid C16:1 in obesity or unsaturated fatty acid C20:4 in obesity/diabetes. Our study provides evidence that the analyses of one “Omics”-like secretome might not be sufficient to get insight in a complex phenotypical problem.

Here, the combination of specific DAG species and the holistic pattern of primary adipocyte-secreted proteins helped to get hints to an interacting mechanism and to unravel RNA-binding proteins involved in metabolic control differing in obesity and obesity/diabetes.

## Figures and Tables

**Table 1:** Top putatively regulated proteins (SP(+), SP(−)) in the comparisons.

Protein Names	Protein ID	Gene Names	BKS_C57	BKS_C57
			Log2 Fold Change	*p*-Value (Welch Test)
Complement factor D	P03953	*Cfd*	−5.42	1.49 × 10^6^
cAMP-dependent protein kinase type II-β	P31324	*Prkar2b*	−2.47	2.73 × 10^6^
Apolipoprotein E	P08226	*Apoe*	−2.17	7.51 × 10^4^
Receptor expression-enhancing protein 6	Q9JM62	*Reep6*	−2.14	9.00 × 10^4^
Lipoprotein lipase	P11152	*Lpl*	−2.03	8.64 × 10^3^
Prolargin	Q9JK53	*Prelp*	−1.90	8.59 × 10^4^
26S protease regulatory subunit 10B	P62334	*Psmc6*	−1.89	6.65 × 10^5^
Coiled-coil domain-containing protein 80	Q8R2G6	*Ccdc80*	−1.89	2.33 × 10^4^
Pentraxin-related protein PTX3	P48759	*Ptx3*	−1.87	3.35 × 10^2^
Tenascin	Q80YX1	*Tnc*	−1.86	6.09 × 10^5^
Inositol polyphosphate 1-phosphatase	P49442	*Inpp1*	1.52	1.24 × 10^5^
Carbonic anhydrase 2	P00920	*Ca2*	1.57	1.29 × 10^4^
Dolichyl-diphosphooligosaccharide-protein glycosyltransferase 48 kDa subunit	O54734	*Ddost*	1.71	9.47 × 10^5^
Dolichyl-diphosphooligosaccharide-protein glycosyltransferase subunit 2	Q9DBG6	*Rpn2*	1.72	4.48 × 10^4^
Nodal modulator 1	Q6GQT9	*Nomo1*	1.78	1.74 × 10^5^
60S ribosomal protein L12	P35979	*Rpl12*	1.86	9.57 × 10^5^
Ketohexokinase	P97328	*Khk*	1.94	9.40 × 10^5^
Carbonyl reductase 3	Q8K354	*Cbr3*	1.98	1.06 × 10^4^
Carbonic anhydrase 1	P13634	*Ca1*	2.38	3.45 × 10^7^
Glutathione S-transferase θ-2	Q61133	*Gstt2*	3.25	4.80 × 10^9^
**Protein Names**	**Protein IDs**	**Gene Names**	**obob_C57**	**obob_C57**
Complement factor D	P03953	*Cfd*	−9.46	5.52 × 10^10^
Collagen α-1(XII) chain	Q60847	*Col12a1*	−6.39	1.12 × 10^9^
Collagen α-5(VI) chain	A6H584	*Col6a5*	−6.20	1.22 × 10^9^
Angiotensinogen	P11859	*Agt*	−4.43	2.77 × 10^7^
Fructose-1,6-bisphosphatase 1	Q9QXD6	*Fbp1*	−3.94	1.45 × 10^7^
Carboxypeptidase Q	Q9WVJ3	*Cpq*	−3.64	1.15 × 10^6^
α-Amylase 1	P00687	*Amy1*	−3.64	1.84 × 10^5^
Coiled-coil domain-containing protein 80	Q8R2G6	*Ccdc80*	−3.47	2.67 × 10^7^
Tissue α-l-fucosidase	Q99LJ1	*Fuca1*	−3.28	9.84 × 10^7^
Ganglioside GM2 activator	Q60648	*Gm2a*	−3.26	2.29 × 10^6^
Actin-related protein 2/3 complex subunit 3	Q9JM76	*Arpc3*	1.94	4.89 × 10^5^
NADH-cytochrome b5 reductase 3	Q9DCN2	*Cyb5r3*	2.07	5.69 × 10^5^
Epoxide hydrolase 1	Q9D379	*Ephx1*	2.12	3.21 × 10^8^
Acyl-CoA dehydrogenase 9, mitochondrial	Q8JZN5	*Acad9*	2.18	5.98 × 10^8^
Serum deprivation-response protein	Q63918	*Sdpr*	2.22	2.03 × 10^6^
Serpin H1	P19324	*Serpinh1*	2.25	3.14 × 10^5^
Galectin-3	P16110	*Lgals3*	2.25	6.50 × 10^7^
GTP:AMP phosphotransferase AK3, mitochondrial	Q9WTP7	*Ak3*	2.28	2.06 × 10^7^
Apolipoprotein A-IV	P06728	*Apoa4*	2.42	6.29 × 10^7^
Interleukin-6	P08505	*Il6*	3.19	6.96 × 10^7^
**Protein Names**	**Protein IDs**	**Gene Names**	**dbdb_BKS**	**dbdb_BKS**
Collagen α-5(VI) chain	A6H584	*Col6a5*	−7.87	3.53 × 10^11^
Fructose-1,6-bisphosphatase 1	Q9QXD6	*Fbp1*	−4.10	1.64 × 10^7^
Tissue α-l-fucosidase	Q99LJ1	*Fuca1*	−3.92	4.32 × 10^8^
Carboxypeptidase Q	Q9WVJ3	*Cpq*	−3.34	1.63 × 10^6^
Complement factor D	P03953	*Cfd*	−3.33	2.71 × 10^4^
Ganglioside GM2 activator	Q60648	*Gm2a*	−3.28	1.18 × 10^6^
Carboxylesterase 1D	Q8VCT4	*Ces1d*	−2.97	1.49 × 10^11^
Dolichyl-diphosphooligosaccharide-protein glycosyltransferase subunit 2	Q9DBG6	*Rpn2*	−2.87	2.22 × 10^4^
Fructose-1,6-bisphosphatase isozyme 2	P70695	*Fbp2*	−2.80	5.61 × 10^5^
Angiotensinogen	P11859	*Agt*	−2.44	2.67 × 10^4^
Phospholipid transfer protein	P55065	*Pltp*	2.77	1.62 × 10^2^
Serum deprivation-response protein	Q63918	*Sdpr*	2.86	5.17 × 10^7^
Polymerase I and transcript release factor	O54724	*Ptrf*	2.93	1.87 × 10^8^
Platelet-activating factor acetylhydrolase	Q60963	*Pla2g7*	2.97	4.03 × 10^3^
Vimentin	P20152	*Vim*	3.12	8.70 × 10^11^
C-C motif chemokine 2	P10148	*Ccl2*	3.37	1.23 × 10^2^
Prolargin	Q9JK53	*Prelp*	3.39	1.65 × 10^6^
cAMP-dependent protein kinase type II-β	P31324	*Prkar2b*	3.70	4.52 × 10^9^
Growth-regulated α protein	P12850	*Cxcl1*	4.36	1.13 × 10^6^
Interleukin-6	P08505	*Il6*	5.35	2.09 × 10^10^
**Protein Names**	**Protein IDs**	**Gene Names**	**dbdb_obob**	**dbdb_obob**
Transthyretin	P07309	*Ttr*	−3.06	9.14 × 10^8^
ATP-citrate synthase	Q91V92	*Acly*	−2.98	4.13 × 10^11^
Sarcosine dehydrogenase, mitochondrial	Q99LB7	*Sardh*	−2.34	3.51 × 10^6^
GTP:AMP phosphotransferase AK3, mitochondrial	Q9WTP7	*Ak3*	−2.15	1.91 × 10^7^
Serpin H1	P19324	*Serpinh1*	−2.01	2.23 × 10^4^
Acyl-CoA dehydrogenase 9, mitochondrial	Q8JZN5	*Acad9*	−1.99	2.95 × 10^7^
Vitamin D-binding protein	P21614	*Gc*	−1.90	9.29 × 10^7^
3-Hydroxyisobutyrate dehydrogenase, mitochondrial	Q99L13	*Hibadh*	−1.86	3.24 × 10^4^
Pyruvate dehydrogenase E1 mitochondrial	P35486	*Pdha1*	−1.81	1.05 × 10^3^
Citrate synthase, mitochondrial	Q9CZU6	*Cs*	−1.70	1.07 × 10^5^
Calmodulin	P62204	*Calm1*	1.51	3.03 × 10^5^
Laminin subunit β-2	Q61292	*Lamb2*	1.55	7.46 × 10^7^
C-X-C motif chemokine 5;GCP-2(1-78);GCP-2(9-78)	P50228	*Cxcl5*	1.77	4.79 × 10^4^
Prolargin	Q9JK53	*Prelp*	1.82	3.83 × 10^3^
Pentraxin-related protein PTX3	P48759	*Ptx3*	1.84	9.82 × 10^3^
Desmin	P31001	*Des*	2.09	5.46 × 10^7^
Lactotransferrin	P08071	*Ltf*	2.15	9.64 × 10^4^
Collagen α-1(XII) chain	Q60847	*Col12a1*	4.45	1.31 × 10^7^

*p*-Value: post hoc test (ANOVA).

**Table 2:** Proteins detected in only one genotype in comparisons.

Protein Names	Protein ID	Gene Names	SP(+)	SP(−)	NP
BKS_C57					
Tyrosine-protein phosphatase non-receptor type 6	P29351	*Ptpn6*			+
NADPH-cytochrome P450 reductase	P37040	*Por*		+	
Vacuolar protein sorting-associated protein 13C	Q8BX70	*Vps13c*			+
Annexin A11	P97384	*Anxa11*		+	
Epidermal growth factor receptor substrate 15	P42567	*Eps15*			+
Adenosine deaminase	P03958	*Ada*			+
Pyruvate dehydrogenase E1 subunit α, mitochondrial	P35486	*Pdha1*		+	
Carnitine *O*-acetyltransferase	P47934	*Crat*		+	
Histidine triad nucleotide-binding protein 1	P70349	*Hint1*		+	
Heat shock protein 75 kDa, mitochondrial	Q9CQN1	*Trap1*		+	
COP9 signalosome complex subunit 8	Q8VBV7	*Cops8*			+
Endothelial cell-selective adhesion molecule	Q925F2	*Esam*	+		
Carbonic anhydrase 4	Q64444	*Ca4*	+		
Arsenite methyltransferase	Q91WU5	*As3mt*			+
Deoxyguanosine kinase, mitochondrial	Q9QX60	*Dguok*		+	
H-2 class I histocompatibility antigen, D-B α chain	P01899	*H2-D1*	+		
Phosphoserine phosphatase	Q99LS3	*Psph*			+
Mannosyl-oligosaccharide 1,2-α-mannosidase IA	P45700	*Man1a1*			+
Band 4.1-like protein 2	O70318	*Epb41l2*			+
Glucosamine-6-phosphate isomerase 1	O88958	*Gnpda1*			+
β-Galactosidase	P23780	*Glb1*	+		
Semaphorin-7A	Q9QUR8	*Sema7a*	+		
Very long-chain acyl-CoA dehydrogenase, mitochondrial	P50544	*Acadvl*		+	
Inter-α-trypsin inhibitor heavy chain H1	Q61702	*Itih1*	+		
Glutathione *S*-transferase θ-1	Q64471	*Gstt1*		+	
Catenin β-1	Q02248	*Ctnnb1*		+	
BTB/POZ domain-containing protein KCTD12	Q6WVG3	*Kctd12*		+	
Retinol-binding protein 2	Q08652	*Rbp2*			+
Small nuclear ribonucleoprotein Sm D3	P62320	*Snrpd3*		+	
Coronin-7	Q9D2V7	*Coro7*			+
DNA topoisomerase 2-β	Q64511	*Top2b*			+
Mast cell protease 2	P15119	*Mcpt2*	+		
Retinol-binding protein 1	Q00915	*Rbp1*			+
Phosphoglycolate phosphatase	Q8CHP8	*Pgp*			+
β-Hexosaminidase subunit β	P20060	*Hexb*	+		
Putative hydroxypyruvate isomerase	Q8R1F5	*Hyi*			+
*S*-adenosylmethionine synthase isoform type-2	Q3THS6	*Mat2a*		+	
Bisphosphoglycerate mutase	P15327	*Bpgm*			+
**obob_C57**					
Semaphorin-7A	Q9QUR8	*Sema7a*	+		
α-Methylacyl-CoA racemase	O09174	*Amacr*			+
Leukemia inhibitory factor	P09056	*Lif*	+		
Fructose-1,6-bisphosphatase isozyme 2	P70695	*Fbp2*		+	
Thiosulfate sulfurtransferase	P52196	*Tst*		+	
Eukaryotic translation initiation factor 3 subunit C	Q8R1B4	*Eif3c*			+
Tyrosine-protein phosphatase non-receptor type 6	P29351	*Ptpn6*			+
Signal transducer and activator of transcription 1	P42225	*Stat1*			+
Fibromodulin	P50608	*Fmod*	+		
Growth-regulated α protein	P12850	*Cxcl1*	+		
Plasminogen activator inhibitor 2, macrophage	P12388	*Serpinb2*		+	
1-Acyl-sn-glycerol-3-phosphate acyltransferase β	Q8K3K7	*Agpat2*	+		
BTB/POZ domain-containing protein KCTD12	Q6WVG3	*Kctd12*		+	
C-X-C motif chemokine 3	Q6W5C0	*Cxcl3*	+		
Epidermal growth factor receptor substrate 15	P42567	*Eps15*			+
26S proteasome non-ATPase regulatory subunit 13	Q9WVJ2	*Psmd13*		+	
Glutathione S-transferase Mu 7	Q80W21	*Gstm7*			+
Hereditary hemochromatosis protein homolog	P70387	*Hfe*	+		
Very long-chain specific acyl-CoA dehydrogenase, mitochondrial	P50544	*Acadvl*		+	
Cadherin-16	O88338	*Cdh16*	+		
C-X-C motif chemokine 5	P50228	*Cxcl5*	+		
α-1-Antitrypsin 1-5	Q00898	*Serpina1e*	+		
**dbdb_BKS**					
Tyrosine-protein phosphatase non-receptor type 6	P29351	*Ptpn6*			+
Hereditary hemochromatosis protein homolog	P70387	*Hfe*	+		
Proteasome activator complex subunit 3	P61290	*Psme3*			+
C-C motif chemokine 9	P51670	*Ccl9*	+		
Tripeptidyl-peptidase 2	Q64514	*Tpp2*			+
Vacuolar protein sorting-associated protein 13C	Q8BX70	*Vps13c*			+
Fibromodulin	P50608	*Fmod*	+		
Eukaryotic translation initiation factor 2 subunit 1	Q6ZWX6	*Eif2s1*			+
Metalloproteinase inhibitor 2	P25785	*Timp2*	+		
Mannosyl-oligosaccharide 1,2-α-mannosidase IA	P45700	*Man1a1*			+
Importin-9	Q91YE6	*Ipo9*		+	
Carnitine *O*-acetyltransferase	P47934	*Crat*		+	
Small glutamine-rich tetratricopeptide repeat-containing protein α	Q8BJU0	*Sgta*		+	
T-complex protein 1 subunit ζ	P80317	*Cct6a*			+
Epidermal growth factor receptor substrate 15	P42567	*Eps15*			+
Arginase-1	Q61176	*Arg1*			+
Granulocyte colony-stimulating factor	P09920	*Csf3*			+
AP-2 complex subunit mu	P84091	*Ap2m1*			+
Phosphoserine phosphatase	Q99LS3	*Psph*			+
Histidine triad nucleotide-binding protein 1	P70349	*Hint1*		+	
Plasminogen activator inhibitor 2, macrophage	P12388	*Serpinb2*		+	
Basigin	P18572	*Bsg*	+		
Dynactin subunit 2	Q99KJ8	*Dctn2*			+
COP9 signalosome complex subunit 8	Q8VBV7	*Cops8*			+
Coatomer subunit ζ-1	P61924	*Copz1*			+
6-Pyruvoyl tetrahydrobiopterin synthase	Q9R1Z7	*Pts*		+	
Inter-α-trypsin inhibitor heavy chain H1	Q61702	*Itih1*	+		
Annexin A11	P97384	*Anxa11*		+	
Plastin-1	Q3V0K9	*Pls1*			+
Eosinophil cationic protein 1	P97426	*Ear1*	+		
Isopentenyl-diphosphate δ-isomerase 1	P58044	*Idi1*			+
Cadherin-1	P09803	*Cdh1*	+		
4-Hydroxy-2-oxoglutarate aldolase, mitochondrial	Q9DCU9	*Hoga1*		+	
Nucleoside diphosphate-linked moiety X motif 19, mitochondrial	P11930	*Nudt19*		+	
Dolichyl-diphosphooligosaccharide-protein glycosyltransferase 48 kDa subunit	O54734	*Ddost*	+		
Thiosulfate sulfurtransferase	P52196	*Tst*		+	
Cysteine and glycine-rich protein 1	P97315	*Csrp1*			+
*N*(*G*),*N*(*G*)-Dimethylarginine dimethylaminohydrolase 1	Q9CWS0	*Ddah1*		+	
Small nuclear ribonucleoprotein Sm D3	P62320	*Snrpd3*		+	
SUMO-conjugating enzyme UBC9	P63280	*Ube2i*		+	
Band 4.1-like protein 2	O70318	*Epb41l2*			+
Semaphorin-7A	Q9QUR8	*Sema7a*	+		
Adenosine deaminase	P03958	*Ada*			+
Coronin-7	Q9D2V7	*Coro7*			+
Nodal modulator 1	Q6GQT9	*Nomo1*	+		
Phosphoglycolate phosphatase	Q8CHP8	*Pgp*			+
C-X-C motif chemokine 3	Q6W5C0	*Cxcl3*	+		
β-Hexosaminidase subunit β	P20060	*Hexb*	+		
Putative hydrolase RBBP9	O88851	*Rbbp9*			+
Ketohexokinase	P97328	*Khk*		+	
Interleukin-1 receptor antagonist protein	P25085	*Il1rn*	+		
α-Methylacyl-CoA racemase	O09174	*Amacr*			+
Protein kinase C δ-binding protein	Q91VJ2	*Prkcdbp*		+	
Retinol-binding protein 1	Q00915	*Rbp1*			+
*S*-adenosylmethionine synthase isoform type-2	Q3THS6	*Mat2a*		+	
Glutathione *S*-transferase θ-2	Q61133	*Gstt2*		+	
Cadherin-16	O88338	*Cdh16*	+		
Bisphosphoglycerate mutase	P15327	*Bpgm*			+
C-X-C motif chemokine 5	P50228	*Cxcl5*	+		
α-1-Antitrypsin 1-5	Q00898	*Serpina1e*	+		
**dbdb_obob**					
Deoxyguanosine kinase, mitochondrial	Q9QX60	*Dguok*		+	
Semaphorin-7A	Q9QUR8	*Sema7a*	+		
Glutathione S-transferase Mu 7	Q80W21	*Gstm7*			+
Phosphoglucomutase-like protein 5	Q8BZF8	*Pgm5*			+
1-Acyl-sn-glycerol-3-phosphate acyltransferase β	Q8K3K7	*Agpat2*	+		
Regulator of microtubule dynamics protein 3	Q3UJU9	*Rmdn3*		+	
Eosinophil cationic protein 1	P97426	*Ear1*	+		
H-2 class I histocompatibility antigen, D-B α chain	P01899	*H2-D1*	+		
Interleukin-1 receptor antagonist protein	P25085	*Il1rn*	+		
Putative hydroxypyruvate isomerase	Q8R1F5	*Hyi*			+

## Supplementary Materials

Supplementary materials can be found at www.mdpi.com/1422-0067/18/9/1928/s1.
